# Cortical Thickness Changes After Computerized Working Memory Training in Patients With Mild Cognitive Impairment

**DOI:** 10.3389/fnagi.2022.796110

**Published:** 2022-04-04

**Authors:** Haakon R. Hol, Marianne M. Flak, Linda Chang, Gro Christine Christensen Løhaugen, Knut Jørgen Bjuland, Lars M. Rimol, Andreas Engvig, Jon Skranes, Thomas Ernst, Bengt-Ove Madsen, Susanne S. Hernes

**Affiliations:** ^1^Department of Radiology, Sørlandet Hospital, Arendal, Norway; ^2^Department of Radiology, Oslo University Hospital, Oslo, Norway; ^3^Department of Clinical Science, University of Bergen, Bergen, Norway; ^4^Department of Pediatrics, Sørlandet Hospital, Arendal, Norway; ^5^Department of Diagnostic Radiology and Nuclear Medicine, University of Maryland School of Medicine, Baltimore, MD, United States; ^6^Department of Neurology, University of Maryland School of Medicine, Baltimore, MD, United States; ^7^Department of Neurology, Johns Hopkins University School of Medicine, Baltimore, MD, United States; ^8^Department of Clinical and Molecular Medicine, Norwegian University of Science and Technology, Trondheim, Norway; ^9^Department of Medicine, Diakonhjemmet Hospital, Oslo, Norway; ^10^Department of Geriatric and Internal Medicine, Sørlandet Hospital, Arendal, Norway

**Keywords:** cortical thickness, MCI, *APOE* genotype, *LMX*1A, working memory training

## Abstract

**Background:**

Adaptive computerized working memory (WM) training has shown favorable effects on cerebral cortical thickness as compared to non-adaptive training in healthy individuals. However, knowledge of WM training-related morphological changes in mild cognitive impairment (MCI) is limited.

**Objective:**

The primary objective of this double-blind randomized study was to investigate differences in longitudinal cortical thickness trajectories after adaptive and non-adaptive WM training in patients with MCI. We also investigated the genotype effects on cortical thickness trajectories after WM training combining these two training groups using longitudinal structural magnetic resonance imaging (MRI) analysis in Freesurfer.

**Method:**

Magnetic resonance imaging acquisition at 1.5 T were performed at baseline, and after four- and 16-weeks post training. A total of 81 individuals with MCI accepted invitations to undergo 25 training sessions over 5 weeks. Longitudinal Linear Mixed effect models investigated the effect of adaptive vs. non-adaptive WM training. The LME model was fitted for each location (vertex). On all statistical analyzes, a threshold was applied to yield an expected false discovery rate (FDR) of 5%. A secondary LME model investigated the effects of *LMX*1A and *APOE-*ε4 on cortical thickness trajectories after WM training.

**Results:**

A total of 62 participants/patients completed the 25 training sessions. Structural MRI showed no group difference between the two training regimes in our MCI patients, contrary to previous reports in cognitively healthy adults. No significant structural cortical changes were found after training, regardless of training type, across all participants. However, *LMX*1A-AA carriers displayed increased cortical thickness trajectories or lack of decrease in two regions post-training compared to those with *LMX*1A-GG/GA. No training or training type effects were found in relation to the *APOE*-ε4 gene variants.

**Conclusion:**

The MCI patients in our study, did not have improved cortical thickness after WM training with either adaptive or non-adaptive training. These results were derived from a heterogeneous population of MCI participants. The lack of changes in the cortical thickness trajectory after WM training may also suggest the lack of atrophy during this follow-up period. Our promising results of increased cortical thickness trajectory, suggesting greater neuroplasticity, in those with *LMX*1A-AA genotype need to be validated in future trials.

## Introduction

Mild Cognitive Impairment (MCI) describes individuals with reduced cognitive function with age not severe enough to meet the criteria of dementia, but more pronounced than the normal age-related cognitive decline reported in healthy controls (Petersen et al., 1997; Langa and Levine, 2014). Individuals with MCI have a 10-fold higher risk of developing dementia compared to healthy adults at the same age (Petersen et al., 1999; Petersen, 2009). Worldwide, the cost of dementia was estimated to be more than 1 trillion USD in 2020 (Prince et al., 2015). Any treatment that can prevent or delay the conversion from MCI to dementia would thus impact the global economy significantly.

Currently, there is no treatment available for MCI, but studies suggest that cognitive training based on the principles of neuroplasticity may aid in delaying the decline in cognitively unimpaired older adults (Smith et al., 2009; Zelinski et al., 2011; Butler et al., 2018). Repeated cognitive stimulation is thought to improve myelination and increase synaptic density in the brain, thereby improving function (Maas and Angulo, 2021). Adaptive computerized cognitive training, with dynamically increased workloads, was found to be effective in improving untrained cognitive functions utilizing the same neural connections as trained tasks (Klingberg et al., 2002). Effects of training may persist for up to 10 years post intervention (Rebok et al., 2014). Studies focusing on the effect of cognitive training in MCI patients have demonstrated improvement of cognitive functions and transfer effects to non-trained domains (Belleville et al., 2006; Talassi et al., 2007; Flak et al., 2019). However, these few studies showed mixed results, possibly due to the heterogeneous phenotypes within the MCI population with various underlying brain pathologies or co-morbid conditions. In addition, the lack of standardized training methods and outcome measures in the field of cognitive training further complicates comparison across studies.

A pair of recent meta-analytic reviews of efficacy of cognitive intervention in individuals with MCI indicates significant overall effects for intervention content, with memory focused interventions appearing to be more effective than multidomain approaches (Sherman et al., 2017; Zhang et al., 2019a). Since reduced working memory (WM) is prevalent in MCI patients and occurs both in the amnestic (memory impaired) and non-amnestic (memory intact) MCI subtypes (Salthouse and Meinz, 1995), focused WM training may be particularly effective. WM is considered to be a core cognitive function and refers to the temporary maintenance of information that is no longer present in the environment for use in ongoing cognition (Nee and D’Esposito, 2018). WM capacity is dependent on a widespread brain network that includes, but not limited to, the supramarginal gyrus, the dorsolateral prefrontal cortex, and medial prefrontal and lateral parietal cortex and the insular bilaterally (Tomasi et al., 2005, 2007; Rottschy et al., 2012), as shown on functional magnetic resonance imaging (MRI) studies.

Furthermore, structural MRI provides a non-invasive *in vivo* approach to visualize the brain changes associated with neuroplasticity beyond theories and models. In healthy middle aged and older adults, Engvig et al. (2010) found increased insular thickness after cognitive training. In addition, increased thickness in the right caudal middle frontal cortex and increased volume of the right pallidum were found in healthy adults only after adaptive computerized WM training, but not after non-adaptive training (Metzler-Baddeley et al., 2016). In contrast, Takeuchi et al. (2011) found decreased thickness both in the frontoparietal region and left temporal superior gyrus after an adaptive multiplication task in a group of students, while Lawlor-Savage et al. (2019) reported no quantitative change after a WM task (N-back) training. Only a few morphometry studies of cognitive training were performed in patients with brain pathologies; no changes after training were found in stroke patients (Nyberg et al., 2018), and a meta review from 2018 found small but supporting evidence of structural neuro plasticity in brain-injured patients after training (Caeyenberghs et al., 2018).

Genetic factors may also impact the effect of cognitive training. Specifically, since dopaminergic function plays an important role in WM and other executive functions (Goldman-Rakic, 1996; Salami et al., 2019), polymorphism of the Lim homeobox transcription factor-alpha (*LMX*1A) gene, which is involved in the maintenance of dopaminergic neurons, was evaluated in relation to WM training (Bellander et al., 2011). Dopaminergic synapses are critical in plasticity (Soderqvist et al., 2012), and reduction in dopaminergic transporters or receptors were related to the effects of aging and cognitive deficits (Chang et al., 2008; Li et al., 2010). Chang et al. (2017) found that functional gain after WM training was greater in patients with HIV-associated neurocognitive disorders who had the *LMX*1A-AA genotype compared to those with the *LMX*1A-GG/GA genotypes. Further, Hernes et al. (2021) found that patients with non-amnestic MCI had greater training gains than a group with amnestic MCI, especially in those with the *LMX*1A-AA genotype.

Another gene that may impact the effects of cognitive training is apolipoprotein epsilon 4 (*APOE*ε4) since having this allele is a potent risk factor for late onset Alzheimer’s disease (Huang and Mucke, 2012). Reduced synaptic plasticity in older adults with *APOE*ε4 carriers (Belleville et al., 2011) may theoretically be related to reduced effect of cognitive training. However, Hernes et al. (2021) found improved WM training gains in MCI patients with the *APOE*ε4 allele, since this allele may demonstrate an antagonistic pleiotropy effect benefiting younger and middle age individuals (Tuminello and Han, 2011; Chang et al., 2016). Similarly, compared to individuals without the *APOE*ε4 allele, the ε4 carriers showed greater compensation, both in magnitude and extent in neuronal activation in the inferior frontal gyrus in the prefrontal cortex during a WM task (Scheller et al., 2017). Nevertheless, *APOE*ε4-carriers with the amnestic type of MCI may not benefit from this allele; in the study by Hernes et al. (2021) amongst the *APOE*ε4-carriers, the amnestic MCI patients showed significant decline in executive function at 16 weeks after the WM training while the non-amnestic MCI patients showed significant improvements. How the *APOE*ε4 allele might impact brain morphometry after WM training is unknown and was explored in the current study.

In this prospective randomized controlled multicenter trial, we investigated the effects of adaptive and non-adaptive WM training on gray matter morphology in individuals with MCI. Based on prior reports, we hypothesized that cortical thickness would increase in regions associated with WM, in particular the prefrontal cortices and the precuneus, after WM training. Furthermore, we explored possible training effects in cortical thickness associated with allelic variations in *APOE*ε4 and *LMX*1A genotypes through secondary analyzes.

## Materials and Methods

### Ethics

The Norwegian Regional Committee for medical and health research ethics, South-Eastern Health region (no: 2013/410) and the Department of Research at each collaborating hospital approved the study registered in ClinicalTrials.gov (NCT01991405).

### Study Design

This is a multicenter randomized controlled double-blind trial (Flak et al., 2014). Individuals with MCI were recruited from the memory clinics at four hospitals in the South-Eastern Health Region of Norway (Sørlandet Hospital Arendal, Telemark Hospital, Oslo University Hospital, and Diakonhjemmet Hospital). The study period was from August 2013 to December 2016.

### Participants/Sample

A total of 461 individuals were diagnosed at the four centers during the study period; 85 of these individuals consented to participate in the current study. The participants were assessed with neuropsychological tests, questionnaires regarding their risk factors, and MRI of the brain as specified by the Norwegian national guidelines established by the Norwegian register of persons assessed for cognitive symptoms (NorCog). Diagnosis of MCI was made in accordance with the Petersen/Winblad criteria for MCI (Petersen, 2004; Winblad et al., 2004). The Socioeconomic status (SES) was assessed with Hollingshead’s index of education and occupational position, scaled from 1 (low) to 5 (high) (Hollingshead and Redlich, 2007).

**FIGURE 1:**
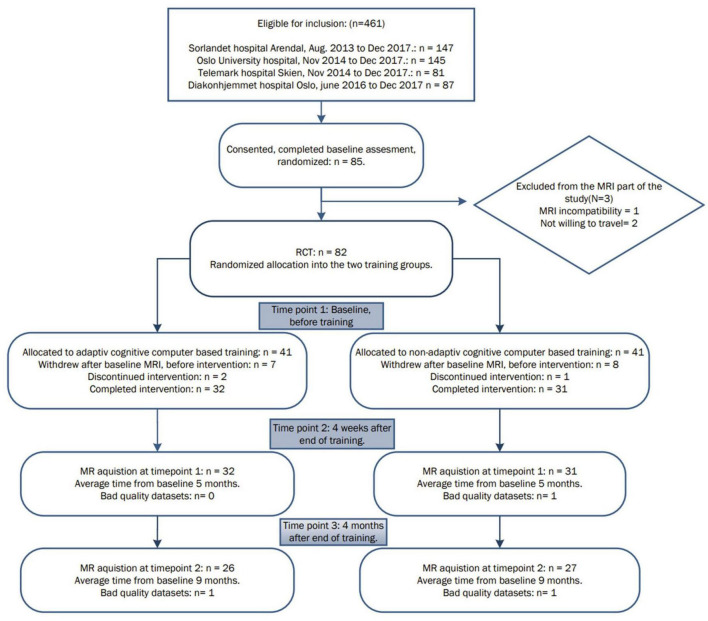
Overview of the patient inclusion process and visits during the study.

The participants underwent neuropsychological assessment and brain MRI as previously described (Flak et al., 2014, 2019). Exclusion criteria included head trauma with a history of post-traumatic brain injury with loss of consciousness, photosensitive epilepsy or any contraindication for MRI (e.g., ferromagnetic metallic implants or severe claustrophobia), use of acetylcholinesterase inhibitors or other antidementia drugs. From the 85 participants enrolled initially, one declined due to MRI contraindications, and two were not willing to travel for the MRI examinations. Eighty-two participants were then randomized for the study; from these participants, 64 completed the training, and 62 had MRI scans from at least two timepoints and were included in the current analysis. Fifty-eight of the 62 participants consented to donate saliva for genetic analysis, but 4 of these participants dropped out of the study and four samples yielded inconclusive results, see Figure 1.

### Cognitive Training

The participants were randomized to either adaptive or non-adaptive cognitive training in accordance with the framework proposed by Simon et al. (2016). The cognitive training was performed at home on the participant’s own computer using the Cogmed WM training program (Klingberg et al., 2005; Klingberg, 2010; Flak et al., 2019). The Cogmed training program consists of several “games” challenging various types of WM. The adaptive training version increases the difficult as the user becomes more skilled, whereas the non-adaptive remains fixed at a low level. The trainers were Cogmed certified, followed the recommended coaching procedures, and monitored the individual participant’s progress continuously *via* reports from the Cogmed software. During training, all participants received phone calls, at least once a week, to follow up on their progress and to motivate them; the calls were made by one of the researchers who followed the participants’ training through an online secured site. Both intervention groups followed the standard protocol (30–40 min of training per day, 5 days per week for 5 weeks). We considered the training completed if the participants finished 20 or more of 25 training sessions (Flak et al., 2019). The different tasks in the program are described in detail in the Supplementary Material.

### Imaging

Magnetic resonance imaging’s were obtained before the initiation of training at baseline (timepoint 1 = tp1), time from cessation of training to second and third image acquisition were kept at similar time intervals; four ± 0.19 weeks after training (timepoint 2 = tp2), and 16 ± 0.72 weeks after training cessation (timepoint 3 = tp3). The total period of follow-up from baseline were 22 ± 1.24 weeks across all participants. Images were acquired on two Siemens 1.5 T Aera scanners (one located in Sorlandet Hospital, Arendal and one at Oslo University Hospital – Rikshospitalet). The MRI setup was identical at both scanners, with the same versions of 20 channel head and neck coils and software (VE11). The MRI sequences included two three-dimensional magnetization-prepared rapid gradient-echo (3D MP-RAGE) scans (sagittal, echo time 3.47 ms, repetition time 2,400 ms, TI 1,000 ms, flip angle 8 degrees, 1.2 mm resolution covering the whole brain). Two test persons were scanned on both machines to assess systematic errors on brain volumes. The participants were scanned on the same scanner at baseline and at each follow-up timepoint. All images were inspected at the MRI console for motion artifacts or other artifacts before the patients were discharged from the MRI facility. Scans with visible motion artifacts were repeated within the same MRI session (Reuter et al., 2015). However, one scan at timepoint 2 and 2 scans at timepoint 3 were discarded due to excessive head motion.

### Postprocessing

Images were converted from DCM image format to Nifti format by the DCM2NIX software.^1^ The FreeSurfer V.6.0^2^ longitudinal processing pathway was used on a small HT condor^3^ computer cluster consisting of three AMD Ryzen© 1800x/1700x CPU equipped workstations, and one AMD Threadripper© 1950x CPU equipped workstation all with ECC memory correction. Cortical reconstruction and segmentation were conducted within the FreeSurfer software suite as described in previous publications (Segonne et al., 2004; Reuter et al., 2010; Reuter and Fischl, 2011).

The longitudinal pathway utilized all the available images for each patient. Twelve participants only had baseline images (tp1) and images 4 weeks after cessation of training (tp2), and 51 participants had MRI images from all three timepoints.

We inspected each scan for skull stripping failure as well as other known errors. The resulting longitudinal cortical thickness parameter was chosen, as it was considered the most reliable for being a surrogate biomarker (Winkler et al., 2018) for a potential WM training effect. Surface maps were resampled to the fsaverage sample provided with Freesurfer, and smoothed with the standard 2D Gaussian smoothing kernel at a value of 30 mm (fwhm) supplied with Freesurfer, as part of the postprocessing procedure, before statistical analysis. Furthermore, the rationale behind the smoothing was to minimize false-positive while still retaining maximum statistical power and counteract some of the anatomical differences between the subjects that the registration process didn’t adapt perfectly and decrease inter-subject variability (Lerch and Evans, 2005; Stelzer et al., 2014; Zeighami and Evans, 2021).

### Genotyping/DNA Collection

Saliva for genotyping was harvested in Oragene Self collection Kit (DAN Genoteck, Inc., Ottawa, ON, Canada) from the participants at study enrolment. Genomic DNA was analyzed with Restriction Fragment Length Polymorphism (RFLP-PCR) for genotype analyzes of *APOE*ε (rs429358 and rs7412) and *LMX*1A (rs4657412), as reported previously (Hernes et al., 2021).

### Statistics

The sample size was calculated on the primary outcome: any group difference in the cortical thickness trajectories. Cohen’s effect size was used to calculate the number of patients for inclusion. To obtain a strong medium effect with Cohen’s effect size of 0.6, approximately 45 patients in either group were needed.

Longitudinal cortical data were analyzed in MATLAB (Mathworks, version 2016a) using a spatiotemporal linear mixed effects model (LME) module supplied with the Freesurfer software. The LME model was fitted for each location (vertex) of the cortical surface (Bernal-Rusiel et al., 2013). To adjust for multiple comparisons, the two p-maps from left and right hemispheres were combined to give equal threshold for both hemispheres. On all statistical analyzes, a threshold was applied to yield an expected false discovery rate (FDR) of 5% (Benjamini et al., 2006) across both hemispheres, to correct for multiple comparisons and prevent false positive results.

First, in order to investigate whether the two groups had similar brain morphometry trajectories over time, a LME model was fitted with cortical thickness as the dependent variable and time (months since first scan), sex, training type (adaptive or non-adaptive), age (at baseline), scanner site (1 at Arendal and 2 at Oslo), and interaction (time × training type) as independent variables. We also used intecept as random factor in all our LME models (Supplementary Table 1).

Cohen’s D for effect size maps were created using a GLM model fitted for each location (vertex), creating maps of the training types’ effect size in a MATLAB model for each timepoint, with the same variables as with the LME model without interaction and random factor.

Secondary analyzes of genotype effects on cortical thickness were conducted on the 50 participants with valid genotype data. Since the two training types showed no group differences on cortical thickness trajectories, the analysis was performed by combining both training types into one group, as we had done in previous analyzes (Hernes et al., 2021). To investigate a possible influence of the *LMX*1A genotype, an LME model was fitted with cortical thickness as dependent variable and time, gender, training type, age, study site, *LMX*1A (AA vs. GG/GA variant), and interaction (time × *LMX*1A genotype) as independent variables, and intercept as random factor. Since only two participants had the GG alleles in the *LMX*1A group, they were combined with the AG alleles group.

To investigate a possible influence of the *APOE*ε gene variants on cortical thickness trajectories, a LME model was fitted with cortical thickness as a dependent variable and time, sex, training type, age, scanner site, presence of *APOE*ε4 allele (ε2/ε2, ε2/ε3, or ε3/ε3 versus ε2/ε4, ε3/ε4 or ε4/ε4), and interaction (time × *APOE*ε gene variants) as independent variables (Chang et al., 2017; Hernes et al., 2021).

### Results

No significant baseline group differences between the two training groups were found for age, sex, socioeconomic status, years of education and full-scale IQ (Table 1). By chance, more of the participants with the *LMX1*A-AA genotype were enrolled in the adaptive training group than the non-adaptive training group.

**TABLE 1:** Clinical characteristics for the adaptive and non-adaptive training groups including genome.

	Adaptive training (*n* = 32)	Non-adaptive training (*n* = 30)	*p*-value	Total (*n* = 62)
	Mean (SD)	Mean (SD)		Mean (SD)
Age (years)^†^	66 (9)	68 (9)	0.326	67 (9)
Sex**^χ2^**	24 Men/8 Women	17 Men/13 Women	0.127	41 Men/21 Women
Socioeconomic status **^χ^^2^**	3.4 (1.19)	3.38 (1.18)	0.934	3.39 (1.18)
Years of education^†^	14 (3)	13 (3)	0.701	14 (3)
Baseline FSIQ^†^	97 (13)	98 (14)	0.772	97 (13)
Valid gene results:	30	24		54
LMX1A genotype (AA or GA/GG) **^χ^^2^**	14 AA (46.7%)/16 GA/GG	5 AA (20.8%)/19 GA/GG	0.014	33 AA (61%)/21 GA/GG
APOE ε (ε4 or non-ε4) **^χ^^2^**	13 ε4 (43,3%)/17 ε3/ε2	11 ε4 (45,8%)/13 ε3/ε2	0.858	23 ε4 (43%)/31 ε3/ε2

*FSIQ, Full Scale Intelligence Quotient; APOE, ε Apolipoprotein e; LMX1A, Lim homeobox transcription factor-alpha; **^χ2^**, Chi Square. ^†^Independent T-test.*

### Adaptive vs. Non-adaptive Training Effects on Cortical Thickness

**FIGURE 2:**
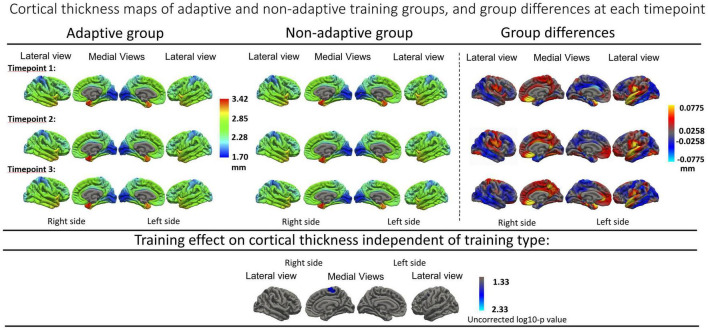
Top panels: Average cortical thickness maps of adaptive and non-adaptive training group, with mean differences from all three timepoints. Timepoint 1: Baseline, timepoint 2: 4 weeks after training cessation. Timepoint 3: 16 weeks after training cessation. Bottom panel: Cortical thickness analysis of training effect independent of training type (time, uncorrected), show no significant region after FDR correction (significance threshold of log10-p: 2.76 = 0.0017). Resampled surface maps, were smoothed with kernel factor of 30 mm (fwhm).

On a linear mixed effects model (Supplementary Table 1), no significant differences were found between the adaptive and non-adaptive training groups in the longitudinal cortical thickness trajectories below the established threshold for FDR correction of 0.000084. The averaged cortical thickness maps for each training group, together with mean cortical thickness difference maps are shown in Figure 2.

**FIGURE 3:**
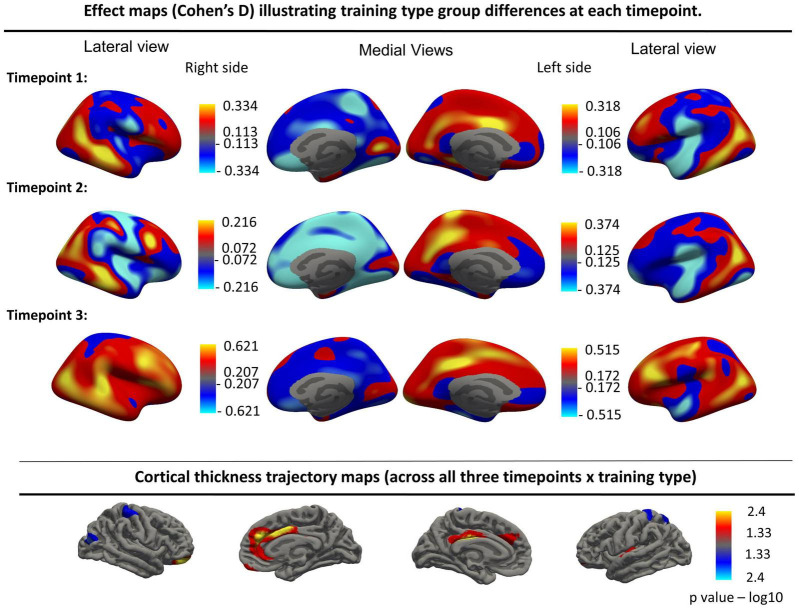
Top panels: Effect maps (Cohen’s D) illustrating training type group differences at each timepoint for the left and right hemispheres. Bottom row: Cortical thickness trajectory maps showing the interaction effect (Time × Training type, uncorrected). These interaction effects were no longer significant after FDR correction. Resampled surface maps, were smoothed with kernel factor of 30 mm (fwhm).

Furthermore, no significant group differences were found when assessing the main effect of time without the interaction term when using a groupwise comparison. Finally, no significant cortical thickness change was found over time when combining the two training type groups. Uncorrected effect sizes expressing longitudinal thickness change between different timepoints in both groups separately are visualized in together with uncorrected cortical trajectory maps in Figure 3.

### LMX1A Genotype Effect on Cortical Thickness After Cognitive Training (Across All Subjects)

**FIGURE 4:**
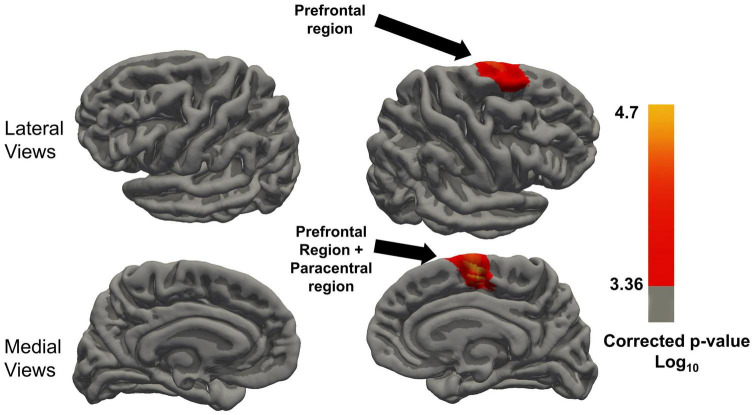
Regions with significant Time x LMX1A (AA vs. GA and GG) interaction on cortical thickness after FDR correction of the *P*-value, minimal significant FDR corrected P threshold is 10^(– 2^.^58)^ = 0.00043. Resampled surface maps, were smoothed with kernel factor of 30 mm (fwhm). Brain regions showing significant group differences (after FDR correction) in the right hemisphere include the superior frontal region, and paracentral region. In the left hemisphere, there was no significant group difference in any region.

In the LME model with the Time*LMX1A interaction, significant differences between the AA and GG/GA carriers were found after FDR correction (corrected threshold 0.00043), which are visualized in Figure 4. Significant clusters of increased cortical thickness trajectory were found in the right superior frontal gyrus, in the AA carriers compared to the GG/GA carriers. In the left hemisphere, the AA carriers showed no different trajectories compared to GG/GA carriers. The mean cortical thickness at each timepoints, and the *p*-values for the interaction effects for the significant clusters per region, and the size of the significant clusters in each region are included in Table 2. No other brain regions showed significant interaction effects, see Figure 4.

**TABLE 2:** Cortical thickness (Mean(±SE)) of regions of interest that show training LMX1A effects, with cluster size of significant vertices.

Timepoint 1			Timepoint 2		Timepoint 3				
Region of interest:	*LMX*1A-AA	*LMX*1A-GA/GG	*LMX*1A-AA	*LMX*1A-GA/GG	*LMX*1A-AA	*LMX*1A-GA/GG	Cluster size of region (%)	Cluster size in mm^2^	Range of corrected *P*-value
Right superior frontal region	2.523 (0.0280)	2.487 (0.0259)	2.508 (0.0318)	2.459 (0.0284)	2.526 (0.0259)	2.472 (0.0282)	21	1438	0.00043–0.000025
Right paracentral region	2.314 (0.02113)	2.316 (0.02269)	2.317 (0.0265)	2.278 (0.0336)	2.344 (0.257)	2.321 (0.0335)	2	30	0.00043–0.000025

*LMX1A, Lim homeobox transcription factor-alpha.*

### *APOE*ε4 Genotype Effect on Cortical Thickness After Cognitive Training (Across All Subjects)

The LME with Time*APOEε4 interaction did not show significant differences in cortical thickness trajectories between the *APOE*ε4 carriers and non-carriers after FDR correction (corrected threshold 0.000126).

## Discussion

This study has the following major findings. Contrary to our hypothesis, cortical thickness did not show significant increase 6 months after WM training in our MCI participants. In addition, the cortical thickness changes were not different between the adaptive and non-adaptive computerized WM training groups. However, a subgroup of MCI participants, those with the *LMX*1A-AA genotype, showed significant increase or lack of decrease of cortical thickness in the right frontal superior region and right paracentral region across WM training groups compared to the GG group. No significant difference in the cortical thickness trajectories after WM training was found between carriers of *APOEε*4 and non-carriers.

Few studies of patients with MCI investigated quantitative changes in brain morphology after cognitive training (Belleville and Bherer, 2012). A recent study by Zhang et al. (2019b) reported a significant correlation between gray matter volume trajectory of the right angular gyrus and the immediate recall component of Hopkins Verbal Learning Test-Revised (HVLT-R) after a multidomain training program in individuals with amnestic MCI. The study is limited by a small sample (*n* = 12) and a non-RCT design. Two studies that evaluated cognitively healthy adults also reported improved cortical thickness after adaptive WM training, but not in those that had non-adaptive training (Metzler-Baddeley et al., 2016; Wu et al., 2021). In contrast, the present study of participants with MCI did not find significant differences in cortical thickness changes between these two types of training. This lack of training type difference on the cortical thickness might have resulted from similar low difficulty levels between the adaptive and non-adaptive training in these MCI patients, that both groups reached a ceiling with regard to the training effect. Currently optimal training time for MCI patients is unknown. Edmonds et al. (2020) reported a 0.05 mm annual atrophy rate in the temporal lobes in individuals with MCI. In our study, no change in the cortical thickness trajectories were observed, which might suggest a lack of decline in cortical thickness after working memory training (WMT). This needs to be explored in further studies.

The lack of group difference on cortical thickness after the two training types may also be due to the study participants’ older age and the heterogeneous etiologies for their MCI (Flak et al., 2019). Therefore, they had limited compensatory processes caused by underlying brain pathologies that might have diminished the training effects on cortical thickness. We speculate that the accumulated degenerative processes in the brains of older MCI individuals might have induced different and less optimal neuroplasticity mechanisms than those present in younger “healthier brains,” leading to the lack of cortical thickness changes after WM training. Some support for this explanation is found in previous reports on task-activated fMRI with greater activity in compensatory brain regions in older adults (Belleville and Bherer, 2012), suggesting a redistribution of the training effects to more widespread brain regions as compared to a more localized effect in younger individuals (Hampstead et al., 2012; Simon et al., 2020).

We further explored the possible genotypic contributions on cortical trajectory changes after WM training since two genotype variants were shown to influence the WM training effects on cognitive outcomes (Hernes et al., 2021). The *LMX*1A gene is involved in the maintenance of dopaminergic neurons, and dopamine is essential for WM function (Puig et al., 2014). Regardless of the WM training type, MCI participants with the *LMX*1A-AA genotype showed significantly increased cortical thickness trajectories after WM training in brain regions associated with WM function. Only one previous longitudinal study has evaluated the effects of WM training on the brain in individuals with the *LMX*1A-AA genotype; Chang et al. (2017) reported decreased BOLD activation on a 2-back fMRI task in those with the AA genotype, suggesting improved neural efficiency, but no change or increased activation in those with GG/GA genotype in the middle frontal gyrus at 1-month after the same WM training. A significant increase in cortical thickness trajectories was observed in the right superior frontal region and continuing over into the paracentral region medially in MCI participants with the *LMX1A*-AA genotype, but not in participants with the GG/GA genotypes. The right superior frontal region is associated with WM functions. Nissim et al. (2016), and a meta review concluded that the superior frontal region was especially sensitive to spatial content (Nee et al., 2012), and right paracentral gyrus was recruited during WM tasks to compensate for having a poor night’s sleep by recruiting the necessary resources to complete the task in a recent neural network study (Lauharatanahirun et al., 2020). Suggesting that it can be involved as a secondary center in WM. Our reported selective increase in cortical thickness trajectory after WM training only in those with the *LMX*1A-AA genotype together with previously published results of increased WM function tests after WM training from the Memory Aid study, suggest that carriers of individuals with *LMX*1A-AA in particular might benefit more from WM training (Hernes et al., 2021) than GG/GA carriers. However, future studies that evaluate this genotypic variant on brain morphometry with a larger sample size are needed.

There were no differences in cortical thickness trajectories among our participant with or without the *APOE-*ε4 allele after WM training. In our previous reports from the same population, *APOEε4* carriers improved in some cognitive tests after WM training as compared to non-carriers (Hernes et al., 2021); therefore, we had expected differential trajectories in cortical thickness changes after WM training. Nevertheless, this negative result should be interpreted with caution due to the relatively small sample size, and further studies with larger sample sizes are needed. To our knowledge, this is the first study that has evaluated the impact of genotypes on brain morphometry after WM training.

### Strengths and Limitations

This study has several strengths. This is a longitudinal follow-up study in a cohort of participants with MCI, which allowed intra-subject assessment of the possible structural brain changes after WM training. Each participant was scanned on the same MRI machines for the baseline and follow-up scans using the identical imaging protocol, thereby minimizing possible variabilities from image contrast, signal-to-noise ratio, contrast-to-noise ratio, intensity non-uniformity or geometric distortion (Lee et al., 2019). Despite these strengths, a limitation to this study is the relatively small sample size for the subgroups, both for comparing the morphological changes between the adaptive and non-adaptive training group, and the comparisons between the morphometric trajectories of the participants with different genotypes. Therefore, these findings should be viewed as preliminary. Furthermore, the manual skull removal that was required for some of the MRI scans might have introduced subjectivity in the early steps of the image processing. Lastly, we also did not evaluate a cognitively healthy control group to determine whether the training effects on cortical thickness might be different between participants with MCI and cognitively healthy individuals. Furthermore the study didn’t include a passive MCI control group, this is in accordance with the framework proposed by Simons et al. (2016) to ensure high-quality computer-based WM training studies.

## Conclusion

The current results from WM training in a heterogeneous population of MCI participants identified the need for further research, especially with respect to genotypic variations in brain neuroplasticity. Promising results of greater neuroplasticity in *LMX*1A-AA carriers should be further investigated in future trials since these individuals may benefit the most from WM training.

## Data Availability Statement

The datasets presented in this article are not readily available because the Norwegian Regional Committee for medical and health research ethics limit data sharing, and de-identified data can only be shared after an application process. The study protocol is publicly available. The statistical analysis plan is available upon request by members of the academic community for the next five years. The generated datasets are available by request to the corresponding author, though no imagesets can be shared, the completed freesurfer data can be requested. Requests to access the datasets should be directed to HH, haakon.ramsland.hol@ous-hf.no.

## Ethics Statement

The studies involving human participants were reviewed and approved by the Norwegian Regional Committee for medical and health research ethics, South-Eastern region (2013/410) approved the study (clinicaltrials.gov NCT01991405). The patients/participants provided their written informed consent to participate in this study.

## Author Contributions

SH, MF, GL, JS, and LC conceptualized and designed the study. MF, GL, AE, B-OM, and HH collected the data. HH, MF, SH, and LC analyzed the data. SH, HH, KB, LR, and LC interpreted the data. HH, SH, KB, AE, LR, and LC drafted the manuscript. All authors have critically revised the article and approved the final version of manuscript to be published.

## Conflict of Interest

The authors declare that the research was conducted in the absence of any commercial or financial relationships that could be construed as a potential conflict of interest.

## Publisher’s Note

All claims expressed in this article are solely those of the authors and do not necessarily represent those of their affiliated organizations, or those of the publisher, the editors and the reviewers. Any product that may be evaluated in this article, or claim that may be made by its manufacturer, is not guaranteed or endorsed by the publisher.

## Footnotes

1
https://github.com/rordenlab/dcm2niix

2
https://surfer.nmr.mgh.harvard.edu/

3
https://research.cs.wisc.edu/htcondor
