# Supplementary material for: Cortical Thickness Changes After Computerized Working Memory Training in Patients With Mild Cognitive Impairment
Source: Front Aging Neurosci. 2022 Apr 4;14:796110. doi: 10.3389/fnagi.2022.796110 (PMC9014119; doi:10.3389/fnagi.2022.796110)
Supplement: Supplementary file 1 [file Table_1.docx]

Supplement figures/tables:

| Selection column: | # |
| --- | --- |
| All participants, even those without gene results. | 1 |
| Either Adaptive trained or non-adaptive trained group | 1b |
| Sub analysis 1: All with Gene results and MRI. | 2 |

The following models were used:

| Variables/Model # | Variables used in the model: |
| --- | --- |
| Model 1a | Intercept(1), Time(years), gender, training groups (randomization), Age from baseline(years), Localization (Arendal (1) or Oslo (2)) and interaction(time x training groups) |
| Model 1.1 | Intercept(1), Time(years), gender, training groups (randomization), Age from baseline(years), Localization (Arendal (1) or Oslo (2)), no interaction term. |
| Model 1.2 | Intercept(1b), Time(years), gender, Age from baseline(years), Localization (Arendal (1) or Oslo (2)), no interaction term. |
| Model 2 | Intercept(2), Time(years), gender, Age from baseline(years),LMX1a gene alleles(AA or AG/GG), localization(Arendal(1) or Oslo(2)) and interaction(time x LMX1a gene alleles). |
| Model 3 | Intercept(2), Time(years), gender, Age from baseline(years), APOE gene variants (E4/EX or E3/E3), localization(Arendal(1) or Oslo(2)) and interaction(time x APOE gene variants). |

 The selection column was also used as the intercept.
